# Recombinant adenovirus expressing vesicular stomatitis virus G proteins induce both humoral and cell-mediated immune responses in mice and goats

**DOI:** 10.1186/s12917-020-02740-6

**Published:** 2021-01-18

**Authors:** Xiaojuan Xue, Zhaorong Yu, Hongyan Jin, Lin Liang, Jiayang Li, Xiaolu Li, Yong Wang, Shangjin Cui, Gang Li

**Affiliations:** 1 Beijing Scientific Observation and Experiment Station for Veterinary Drugs and Diagnostic Technology, Ministry of Agriculture and Rural Affairs, China /Institute of Animal Sciences, Chinese Academy of Agricultural Sciences, Beijing, 100193 China; 2 Anhui Province Key Laboratory of Veterinary Pathobiology and Disease Control, College of Animal Science and Technology, Anhui Agricultural University, Hefei, 230036 China; 3 Tibet Vocational Technical College, Lhasa, 850000 China

**Keywords:** Vesicular stomatitis virus, Recombinant adenovirus, Immune responses, Mice, Goats

## Abstract

**Background:**

Vesicular stomatitis (VS) is an acute, highly contagious and economically important zoonotic disease caused by the vesicular stomatitis virus (VSV). There is a need for effective and safe stable recombinant vaccine for the control of the disease. The human type 5 replication-defective adenovirus expression vector is a good way to construct recombinant vaccines.

**Results:**

Three recombinant adenoviruses (rAd) were successfully constructed that expressed the VSV Indiana serotype glycoprotein (VSV-IN-G), VSV New Jersey serotype glycoprotein (VSV-NJ-G), and the G fusion protein (both serotypes of G [VSV-IN-G-NJ-G]) with potentiality to induce protective immunity. G proteins were successfully expressed with good immunogenicity. The rAds could induce the production of VSV antibodies in mice, and VSV neutralizing antibodies in goats, respectively. The neutralizing antibody titers could reach 1:32 in mice and 1:64 in goats. The rAds induced strong lymphocyte proliferation in mice and goats, which was significantly higher compared to the negative control groups.

**Conclusions:**

The three rAds constructed in the study expressed VSV-G proteins and induced both humoral and cellular immune responses in mice and goats. These results lay the foundation for further studies on the use of rAds in vaccines expressing VSV-G.

**Supplementary Information:**

The online version contains supplementary material available at 10.1186/s12917-020-02740-6.

## Background

Vesicular stomatitis (VS) is a highly contagious and economically important disease of cattle, horses, pigs, and other mammals having zoonotic significance [1, 2]. The etiology of the disease is vesicular stomatitis virus (VSV), a RNA virus of the family *Rhabdoviridae* [3]. The characteristics features of the disease include vesicular lesions in the mouth (lips, gums, tongue), nostrils, coronary band, and teats [4]. Although, no longer listed by the World Organization for Animal Health (OIE), VS is an important disease due to its considerable economic impact on equine events and the fact that it is clinically indistinguishable from foot-and-mouth disease (FMD) [3, 5, 6]. VSV has two main serotypes i.e.*,* Indiana (VSV-IN) and New Jersey (VSV-NJ) that are morphologically and pathologically similar, but generate distinct neutralizing antibodies in infected animals. These virus can be identified by neutralization and complement fixation tests [7, 8]. Although these two serotypes are morphologically and pathologically similar, in infected animals they generate distinct neutralizing antibodies [9]. The cross-protection between the two serotypes is poor; thus, different vaccines are required for each serotype [10, 11]. From 2004 to 2006, 751 VS outbreaks caused by VSV-NJ were reported in the US [12]. These VS outbreaks also spread from the US to Mexico from 2005 to 2011; however, no new variant strain was produced [13]. As VS can be prevented and controlled by vaccination, some advancements have been made with regard to developing vaccines against VSV. However, further efforts are still needed in developing new vaccines [10, 11, 14].

The recombinant adenovirus (rAd) vector has been widely used to achieve transient inducible expression [15]. The adenovirus vector can either be replication-defective or replicative [16]. In the replication-defective adenovirus vector, all structural genes are removed, and only the vital genes for cis-acting elements and the packaging signal sequence are retained; therefore, its cytotoxicity is significantly reduced. Foreign sequences of up to 37 Kb can be inserted into this vector. In addition, the cytomegalovirus (CMV) promoter is also inserted into this vector to enable the efficient expression of foreign genes. This expression system is currently the main vector used for rAd vector vaccine research [17].

The vesicular stomatitis virus glycoprotein (VSV-G) contains glycosylation sites and the antigenic determinant [18]. The VSV-G present as protrusions on the virus envelope is strain-specific and capable to stimulate the production of neutralizing antibodies. VSV-G is the preferred antigen for VSV vaccine research [19]. Effective vaccines currently available against VSV are mostly live attenuated. Since attenuated virus has the risks of virulence reversion, a stable vaccine is therefore in need. In this study we assessed the potential of the VSV-G gene as a vaccine candidate by using a replication-defective human adenovirus type 5 expression vector expressing the VSV-IN glycoprotein (VSV-IN-G), VSV-NJ glycoprotein (VSV-NJ-G), and the G fusion protein (both serotypes of G [VSV-IN-G-NJ-G]). Furthermore, we evaluated the immunogenicity of these rAd vectors expressing VSV-G in mice and goats.

## Results

### Generation and identification of recombinant adenovirus

**Fig. 1:**
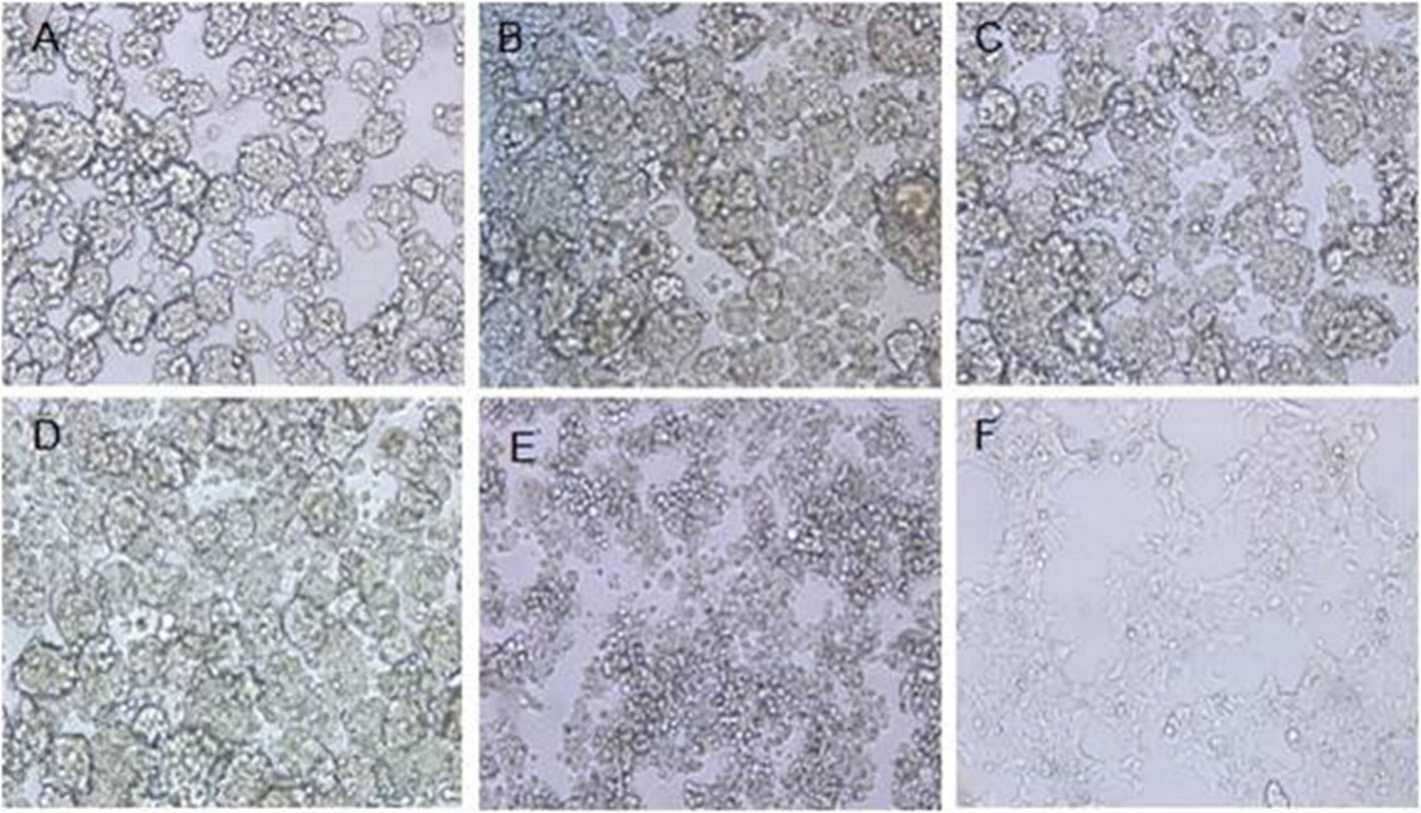
CPE of AAV-293 cells infected with the recombinant adenoviruses. A: CPE of AAV-293 cells infected with rAd-IN; B: CPE of AAV-293 cells infected with rAd-NJ; C: CPE of AAV-293 cells infected with rAd-IN-NJ; D: CPE of AAV-293 cells infected with wtAd; E: CPE of AAV-293 cells infected with VSV-IN; F: Uninfected AAV-293 cells. CPE, cytopathic effect; rAd-IN, recombinant adenovirus-Indiana, rAd-NJ, recombinant adenovirus-New Jersey; rAd-IN-NJ, recombinant adenovirus-Indiana-New Jersey; wtAd, wild-type adenovirus; VSV-IN, vesicular stomatitis virus-Indiana

**Fig. 2:**
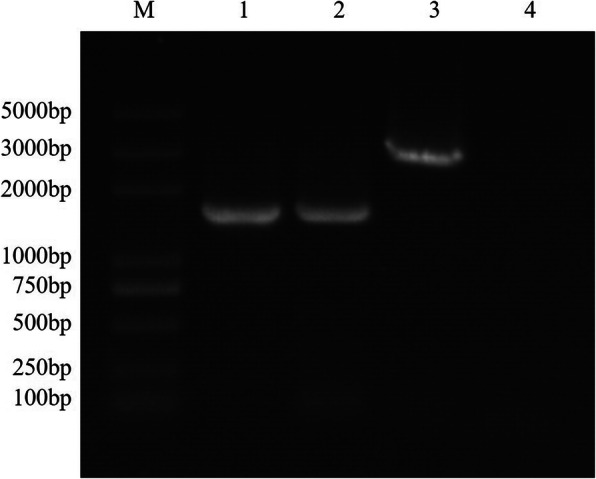
Identification of the recombinant adenovirus by PCR. M: 2 K Plus DNA marker; 1: PCR product from rAd-IN; 2: PCR product from rAd-NJ; 3: PCR product from rAd-IN-NJ; 4: Negative control. rAd-IN, recombinant adenovirus-Indiana; rAd-NJ, recombinant adenovirus-New Jersey; rAd-IN-NJ, recombinant adenovirus-Indiana-New Jersey; PCR, polymerase chain reaction

The three rAds named rAd-IN, rAd-NJ, rAd-IN-NJ were replicated in AAV-293 cells. After a 10-day incubation period, the cytopathic effect (CPE) was observed in the infected cells (Fig. 1). The three rAds were serially propagated to four generation and the rAds were collected to extract the viral DNA. The VSV-IN-G gene (1536 bp), VSV-NJ-G gene (1554 bp) and the VSV-IN-G-NJ-G gene (3100 bp) were amplified by PCR using specific primers (Fig. 2).

### Production and characterization of recombinant adenovirus

**Fig. 3:**
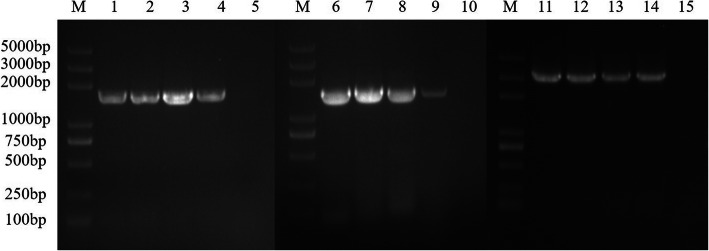
PCR identification of different passages the recombinant adenovirus. M: 2 K Plus DNA marker; 1–4: PCR product from rAd-IN at the 5th, 10th, 15th, and 20th passage; 6–9: PCR product from rAd-NJ at the 5th, 10th, 15th, and 20th passage; 11–14: PCR product from rAd-IN-NJ at the 5th, 10th, 15th, and 20th passage; 5, 10, 15: Negative control. rAd-IN, recombinant adenovirus-Indiana; rAd-NJ, recombinant adenovirus-New Jersey; rAd-IN-NJ, recombinant adenovirus-Indiana-New Jersey; PCR, polymerase chain reaction

The three rAds were inoculated into the AAV-293 cells and were serially propagated to 20 generations. The 5th, 10th, 15th, and 20th generation rAds were collected to extract the viral DNA, and the VSV-IN-G gene (1536 bp), VSV-NJ-G gene (1554 bp) and the VSV-IN-G-NJ-G gene (3100 bp) were then correctly identified using PCR and DNA sequencing, which matched the expected results (Fig. 3). No band was observed in the control group. The TCID_50_’s of the 5th, 10th, 15th, and 20th rAd-IN generations were 10^–8.56^/mL, 10^–8.16^/mL, 10^–8.96^/mL, and 10^–8.34^/mL, respectively. The TCID_50_’s of the 5th, 10th, 15th, and 20th rAd-NJ generation were 10^–7.76^/mL, 10^–8.16^/mL, 10^–7.67^/mL, and 10^–8.2^/mL respectively, and the corresponding values for rAd-IN-NJ were 10^–6.96^/mL, 10^–7.16^/mL, 10^–7.67^/mL, and 10^–7.2^/mL suggesting certain level of genetic stability of the rAds.

### Detection of protein expression by western blotting analyses and immunofluorescence assay

**Fig. 4:**
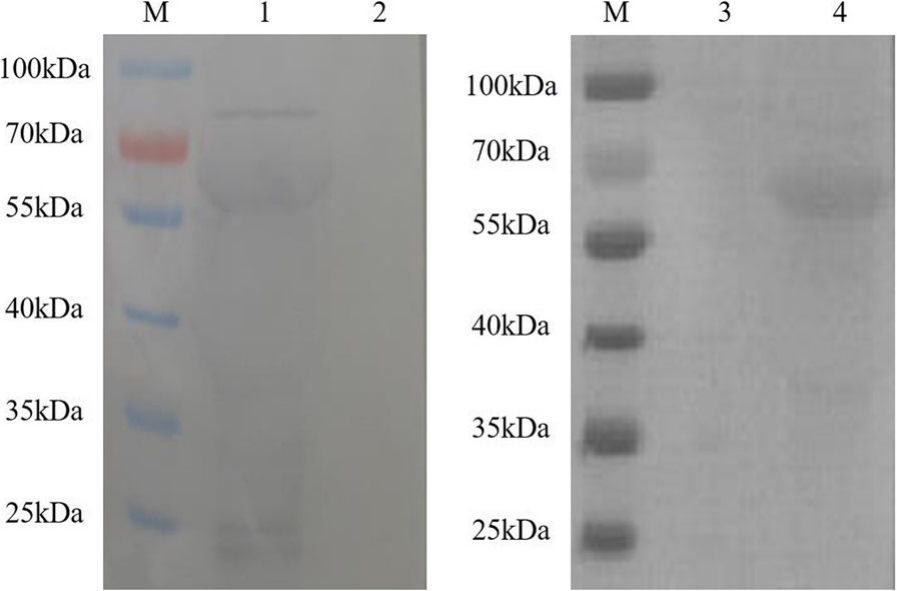
Identification of the G proteins of rAd-IN and rAd-NJ in AAV- 293 cells by western blot. M: Protein molecular weight marker; 1: AAV-293 cells infected with rAd-IN (detected by the VSV-IN G monoclonal antibody); 2: AAV-293 cells infected with wtAdV (detected by the VSV-IN G monoclonal antibody); 3: AAV-293 cells infected with wtAdV (detected by the VSV-NJ polyclonal antibody); 4: AAV-293 cells infected with rAd-NJ (detected by the VSV-NJ polyclonal antibody). rAd-IN, recombinant adenovirus-Indiana; rAd-NJ, recombinant adenovirus-New Jersey; wtAd, wild-type adenovirus

**Fig. 5:**
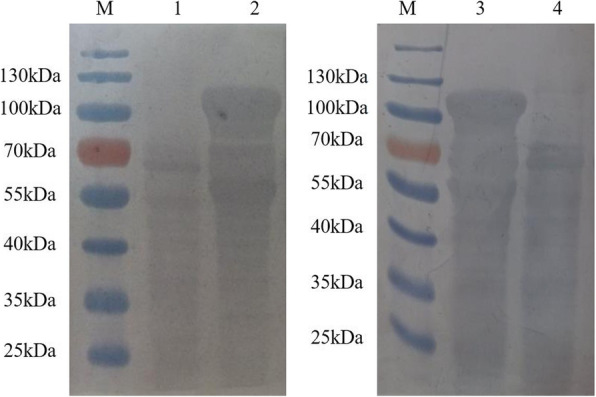
Identification of the fusion G protein of rAd-IN-NJ in AAV-293 cells using a Western blotting. M: Protein molecular weight marker; 1: AAV-293 cells infected with wtAd (detected by the VSV-IN G monoclonal antibody); 2: AAV-293 cells infected with rAd-IN-NJ (detected by the VSV-IN G monoclonal antibody); 3: AAV-293 cells infected with rAd-IN-NJ (detected by the VSV-NJ polyclonal antibody); 4: AAV-293 cells infected with wtAd. wtAd, wild-type adenovirus; rAd-IN-NJ, recombinant adenovirus-Indiana-New Jersey.

The rAd-infected AAV-293 cells were assessed by western blotting to detect the expression of VSV-G. On the blots, the presence of 57 kDa, 57 kDa, and 114 kDa bands which corresponded to VSV-IN-G, VSV-NJ-G, and VSV-IN-G-NJ-G, respectively, were observed (Figs. 4 and 5). Furthermore, rAd-IN and rAd-NJ were detected by the antibodies of the corresponding serotypes. Since the rAd-IN-NJ contained both the VSV-IN-G and VSV-NJ-G genes, both anti-VSV-IN-G protein mouse McAb and anti-VSV-NJ goat PcAb were used to detect the fusion protein.

**Fig. 6:**
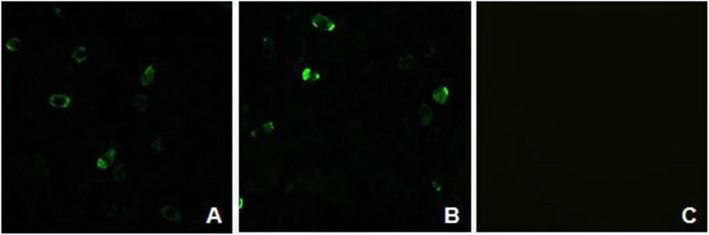
Identification of the fuson G protein of rAd-IN-NJ in Vero cells using an immunofluorescence assay. A: Vero cells infected with rAd-IN-NJ (detected by the VSV-IN monoclonal antibody); B: Vero cells infected with rAd-IN-NJ (detected by the VSV-NJ polyclonal antibody); C: Vero cells infected with wtAd control (detected by the VSV-IN monoclonal or VSV-NJ polyclonal antibody). rAd, recombinant adenovirus; wtAd, wild-type adenovirus; rAd-IN-NJ, recombinant adenovirus-Indiana-New Jersey; VSV-IN, vesicular stomatitis virus-Indiana; VSV-NJ, vesicular stomatitis virus-New Jersey

Vero cells were infected with rAd-IN-NJ to detect the expression of VSV-G by immunofluorescence assay **(**IFA). IFA showed that specific green fluorescence was observed for rAd-IN-NJ, which was not detected in the negative control wild-type Ad (wtAd) (Fig. 6). These results demonstrated that the recombinant fusion G protein could be expressed in-vitro following rAd infection in Vero cells, and that the protein could retain its antigenic reactivity.

### Virus neutralizing antibody responses

The mice and goats immunized with rAds containing the VSV-G genes using a micro-neutralization assay suggested that these animals could produce neutralizing antibodies after immunization. The virus-neutralizing antibody (VNA) titers in all the experimental groups were low after the first immunization in mice and goats, but these titers significantly increased after the second immunization. As shown in Tables 1 and 2, VNA was detected in mice and goats after immunization with rAd-IN or rAd-IN-NJ, and the VNA titers with VSV-IN ranged from 1:8 to 1:32 after the secondary immunization. Furthermore, as shown in Tables 3 and 4, the VNA titers with VSV-NJ were equal to or exceeded a ratio of 1:8 after the second immunization in the rAd-NJ or rAd-IN-NJ vaccinated mice and goat groups. In the neutralization assay with VSV-IN, the VNA titer of the rAd-NJ group was lower than those of the rAd-IN or rAd-IN-NJ vaccinated mice and goat groups. Upon conducting the neutralization assay with VSV-NJ, the VNA titers of the rAd-NJ and rAd-IN-NJ groups were higher than that of the rAd-IN vaccinated mice and goat groups. As expected, no neutralization activity against VSV was detected in the pre-immune sera or in the sera of experimental animals injected with wtAd and phosphate-buffered saline (PBS).

**Table 1:** Detection of VSV neutralizing antibodies in mice induced by recombinant adenovirus with VSV-IN at different time points

Group	Mean titers over time after first immunization^a^			
	0 weeks	2 weeks	4 weeks	6 weeks
rAd-IN	0	< 2 (0–2)	10.4 (8–16)	24 (16–32)
rAd-NJ	0	< 2 (0–2)	6 (4–8)	10.4 (8–16)
rAd-IN-NJ	0	< 2 (0–2)	24 (16–32)	28.8 (16–32)
wtAd	0	0	0	0
PBS	0	0	0	0

^a^Mean neutralizing antibody titers at that time point (range of antibody titers). *VSV-IN* vesicular stomatitis virus-Indiana; *rAd-IN* recombinant adenovirus-Indiana; *rAd-NJ* recombinant adenovirus-New Jersey; *rAd-IN-NJ* recombinant adenovirus-Indiana-New Jersey; *wtAd* wild-type adenovirus; *PBS* phosphate-buffered saline

**Table 2:** Detection of VSV neutralizing antibodies in goats induced by recombinant adenovirus with VSV-IN at different time points

Group	Mean titers over time after first immunization^a^				
	0 weeks	3 weeks	6 weeks	9 weeks	12 weeks
rAd-IN	0	7 (4–8)	28 (16–32)	56 (32–64)	28 (16–32)
rAd-NJ	0	7 (4–8)	14 (8–16)	14 (8–16)	10 (8–16)
rAd-IN-NJ	0	7 (4–8)	14 (8–16)	24 (16–32)	20 (16–32)
wtAd	0	0	0	0	0
PBS	0	0	0	0	0

^a^Mean neutralizing antibody titers at that time point (range of antibody titers). *VSV-IN* vesicular stomatitis virus-Indiana; *rAd-IN* recombinant adenovirus-Indiana; *rAd-NJ* recombinant adenovirus-New Jersey; *rAd-IN-NJ* recombinant adenovirus-Indiana-New Jersey; *wtAd* wild-type adenovirus; *PBS* phosphate-buffered saline

**Table 3:** Detection of VSV neutralizing antibodies in mice induced by recombinant adenovirus with VSV-NJ at different time points

Group	Mean titers over time after first immunization^a^			
	0 weeks	2 weeks	4 weeks	6 weeks
rAd-IN	0	< 2 (0–2)	6 (4–8)	8 (8)
rAd-NJ	0	< 2 (0–2)	10.4 (8–16)	24 (16–32)
rAd-IN-NJ	0	< 2 (0–2)	12 (8–16)	27.2 (16–32)
wtAd	0	0	6 (4–8)	8 (8)
PBS	0	0	10.4 (8–16)	24 (16–32)

^a^Mean neutralizing antibody titers at that time point (range of antibody titers). *VSV-NJ* vesicular stomatitis virus-New Jersey; *rAd-IN* recombinant adenovirus-Indiana; *rAd-NJ* recombinant adenovirus-New Jersey; *rAd-IN-NJ* recombinant adenovirus-Indiana-New Jersey; *wtAd* wild-type adenovirus; *PBS* phosphate-buffered saline

**Table 4:** Detection of VSV neutralizing antibodies in goats induced by recombinant adenovirus with VSV-NJ at different time points

Group	Mean titers over time after first immunization^a^				
	0 weeks	3 weeks	6 weeks	9 weeks	12 weeks
rAd-IN	0	5 (4–8)	10 (8–16)	10 (8–16)	12 (8–16)
rAd-NJ	0	6 (4–8)	20 (16–20)	47 (32–64)	20 (16–32)
rAd-IN-NJ	0	6 (4–8)	18 (8–18)	47 (32–64)	24 (16–24)
wtAd	0	0	0	0	0
PBS	0	0	0	0	0

^a^Mean neutralizing antibody titers at that time point (range of antibody titers). *VSV-NJ* vesicular stomatitis virus-New Jersey; *rAd-IN* recombinant adenovirus-Indiana; *rAd-NJ* recombinant adenovirus-New Jersey; *rAd-IN-NJ* recombinant adenovirus-Indiana-New Jersey; *wtAd* wild-type adenovirus; PBS, phosphate-buffered saline

### Lymphocyte proliferation response

**Fig. 7:**
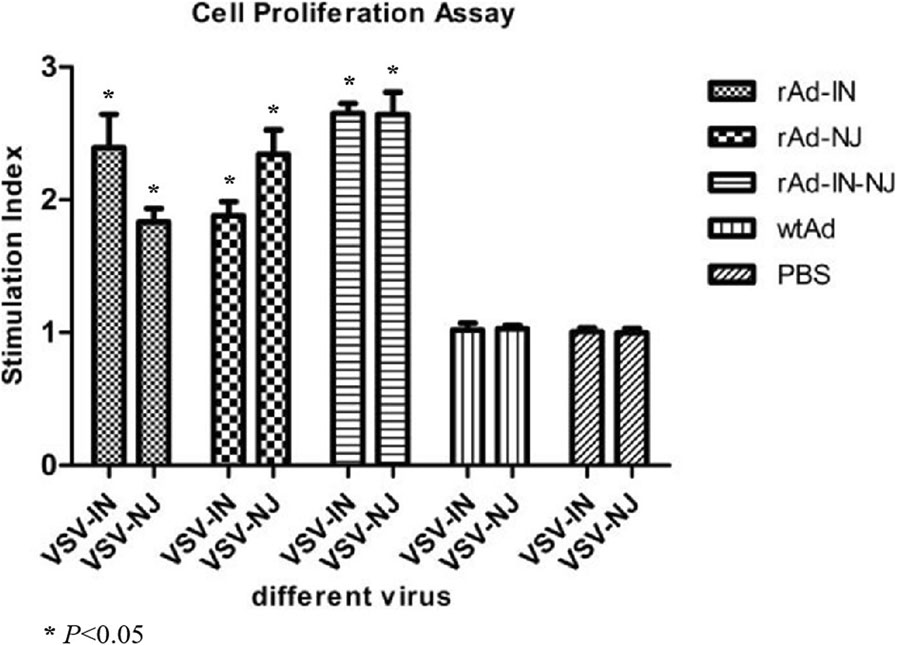
Proliferation of splenocytes to virus stimulation in the immunized mice. rAd-IN, recombinant adenovirus-Indiana; rAd-NJ, recombinant adenovirus-New Jersey; rAd-IN-NJ, recombinant adenovirus-Indiana-New Jersey; wild-type adenovirus; PBS, phosphate-buffered saline; VSV-IN, vesicular stomatitis virus-Indiana; VSV-NJ,vesicular stomatitis virus-New Jersey

**Fig. 8:**
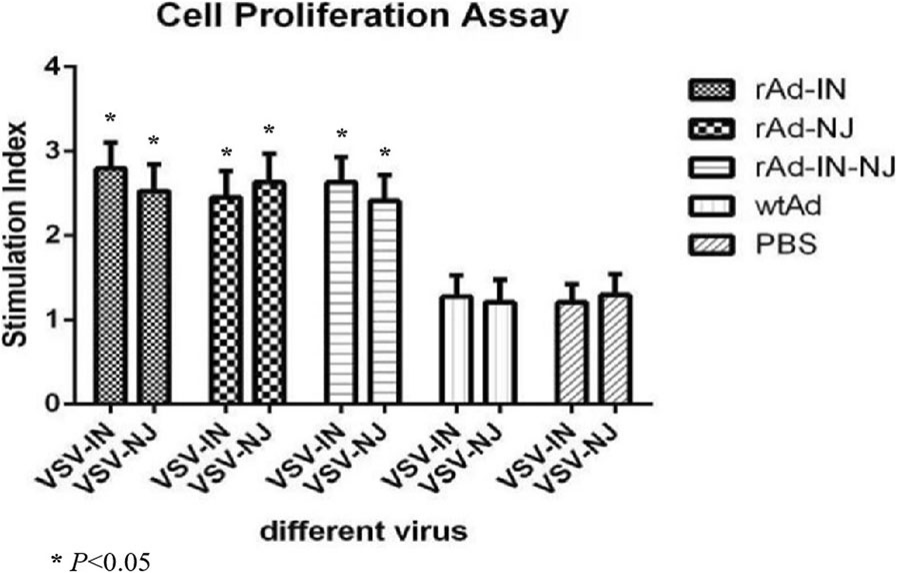
Proliferation of peripheral blood mononuclear cells to virus stimulation in the immunized goats. rAd-IN, recombinant adenovirus-Indiana; rAd-NJ, recombinant adenovirus-New Jersey; rAd-IN-NJ, recombinant adenovirus-Indiana-New Jersey; wild-type adenovirus; PBS, phosphate-buffered saline; VSV-IN, vesicular stomatitis virus-Indiana; VSV-NJ, vesicular stomatitis virus-New Jersey

To investigate the cell-mediated immune responses induced by the three rAds, the lymphocyte proliferation responses of mice were analyzed 2 weeks after the third immunization. In case of goats, the lymphocyte proliferation responses were analyzed 6 weeks after the secondary immunization. VSV-IN and VSV-NJ are two different antigens that stimulated lymphocyte proliferation in different experimental groups. The data showed (Fig. 7 and 8) that mice and goats immunized with the rAds induced significantly stronger lymphocyte proliferation responses by VSV-IN and VSV-NJ compared with the PBS and wtAd control groups. When stimulated by VSV-IN, the lymphocyte proliferative responses in the rAd-IN and rAd-IN-NJ groups were stronger, with a stimulation index (SI) of approximately 2.5. In addition, the SI of the rAd-IN-NJ group was relatively higher than those of the rAd-IN and rAd-NJ groups in mice and goats. There was a significant difference between the rAd groups and the PBS and wtAd control groups (*P* < 0.05), but there was no significant difference among mice immunized with the different rAds (*P* > 0.05).

## Discussion

Vesicular stomatitis is a viral disease that primarily affects horses, cattle, and swine. The virus is capable to affect a wide range of host including sheep and goats. Thus, effective vaccination is very important for the prevention and control of VS. Currently, controlling the spread of VS involves isolation of the affected animals, quarantining the affected premise and is mainly dependent on the immunization of susceptible animals. In addition to inactivated vaccines, live attenuated vaccines are widely used for immunization against VSV. However, due to the risk of virulence reversion in such vaccines, heat-stable, safe, and effective VS vaccines need to be developed.

The human type 5 replication-defective adenovirus expression vector is a common vector used in the research of vaccines and genetic therapy. The newly developed heat-stable technology enables adenovirus vector vaccines to be stored at room temperature up to 45 °C for 6 months and maintain a steady infection hold. rAd vector vaccines can be inoculated using a variety of methods since they have a wider range of permissive host cells. As effective vaccine vectors both the replicative and non-replicative adenovirus are long been used to infect a wide varieties of mammalian cells to to expression higher level of recombinant proteins [20–25]. Considering these advantages, it is valuable to perform further research for the development of vaccine candidates using an adenovirus vector for the prevention and control of VS.

VSV glycoprotein plays major role in the mechanism of disease production in in many of the natural host including pigs [26]. Therefore, in this study we targeted VSV glycoproteingenes to clone and induce with an aim to induce VSV-G proteins for better immune response. It has been reported that the recombinant VSV-IN containing VSV-NJ-G-IN-G expressed both glycoproteins stably through multiple rounds of replication in pigs and induced neutralizing antibodies against both VSV serotypes [10, 26]. In our study, the genes for both VSV-G serotypes were accurately cloned by reverse transcription-PCR (RT-PCR), and the fusion VSV-IN-G-NJ-G gene of 3100 bp was successfully amplified by overlapping PCR using the GlyGlyGlyGlySer polypeptides. In order to determine the best time to harvest the rAds from the cell culture, we performed numerous rAd optimization experiments, and found that 10–15 days was the appropriate time to harvest the rAds. In addition, PCR was conducted to amplify the target genes from the cell culture supernatants containing rAds to confirm the genetic stability of the VSV-G genes. The western blotting and IFA results suggested that the three target proteins could be expressed in AAV-293 cells and Vero cells, but that rAd could only induce a CPE in AAV-293 cells at 36–48 h after virus infection.

One of the major aim of the study was to evaluate the immunogenicity of the newly constructed rAds. Mice and goats inoculated with rAd-IN, rAd-NJ, or rAd-IN-NJ were subjected to virus neutralization test to detect neutralizing antibody levels in their sera at different time interval. The results of the lymphocyte proliferation test showed that the rAds could elicit lymphocyte proliferation in mice and goats, and was significantly higher compared to the negative control groups (*P* < 0.05).

## Conclusions

The newly constructed rAds efficiently expressed the foreign proteins (VSV-IN-G, VSV-NJ-G, and VSV-IN-G-NJ-G). In immunized mice and goats these expressed proteins were able to induce certain degree of both a humoral and cellular immune responses. Mice and goats may be used as model animals to evaluate the VSV vaccine through challenge studies.

## Methods

### Ethical permission

All experiments were approved by the Ethics committee of Institute of Animal Sciences, Chinese Academy of Agricultural Sciences (permit number: 2015–014). All manipulations were carried out in accordance with the requirements of the Regulations of Experimental Animal Administration of China.

### Cells and viruses

AAV-293 and Vero cells were cultured in Dulbecco’s Modified Eagle’s Medium (DMEM) containing 10% fetal bovine serum (FBS), 100 IU/mL penicillin, and 100 IU/mL streptomycin at 37 °C in 5% CO_2_. VSV-IN and VSV-NJ stocks were propagated and titrated in Vero cells.

### Experiment animals

6–8-week-old female BALB/c mice were purchased from the Chinese Experimental Animal Resources Research Institute for Food and Drug Control (Beijing, China). Outbred healthy Boer goats (6 months old) were purchased from local self-supporting farmers in Fuyang, China.

### Construction of plasmids and recovery of recombinant viruses

The VSV-IN-G gene was amplified by reverse transcription-PCR (RT-PCR) from the purified viral (VSV-IN) RNA using the following two primers: 5′-CGGAATTC_*Eco*R I_GCCACC_(kozak sequence)_ATGAAGTGCCTTTTGTA-3′ and 5′-ATTTGCGGCCGC_*Not* I_TTACTTTCCAAGTCGGTTC-3′.

The VSV-NJ-G gene was amplified by RT-PCR from the purified viral (VSV-NJ) RNA using the following primers: 5′-CGGAATTC_*Eco*R I_GCCACC_(kozak sequence)_ATGTTGTCTTATCTAATCTTTGCA-3′ and 5′-ATTTGCGGCCGC_*Not* I_TTAACGGAAATGAGCCATTTCCACG-3′. The gene for the VSV-IN-G-NJ-G fusion protein was amplified by overlapping PCR of polypeptides Gly-Gly-Gly-Gly-Ser and the following two primers (containing the linkers´ gene sequence): 5′-GGTGGAGGTGGAAGC_linker_ATGTTGTCTTATCTAATC-3′ and 5′-GCTTCCACCTCCACC_linker_CTTTCCAAGTCGGTTC-3′.

The target genes (VSV-IN-G, VSV-NJ-G, VSV-IN-G-NJ-G) and the adenovirus shuttle vector (pacAd-CMV K-NpA) were digested with restriction enzymes *Eco*R I and *Not* I. Then, the fragments were cloned into the shuttle vector and ligated with the T4 DNA ligase to construct the recombinant shuttle plasmids containing the target genes (pAd-VSV-IN-G, pAd-VSV-NJ-G, pAd-VSV-IN-G-NJ-G). The recombinant shuttle plasmids and adenovirus backbone plasmids linearized with *Pac*I restriction enzymes were co-transferred into an AAV-293 cell monolayer in six-well tissue culture plates (Costar, Corning, NY, USA) using Lipofectamine-2000 reagent (Invitrogen, USA) as per manufacturer’s instruction. The three rAds constructed in this study were named rAd-IN, rAd-NJ, and rAd-IN-NJ.

The three rAds inoculated into the AAV-293 cells were serially propagated to 20 generations. The CPE was observed and recorded 3 days post-incubation, and TCID_50_ was calculated using the Reed-Muench method.

### Detection of expressing G protein by western blotting and IFA

AAV-293 cells were infected with rAd-IN, rAd-NJ and rAd-IN-NJ. Mouse anti-VSV-IN-G protein McAb, goat anti-VSV-NJ-G protein PcAb were used to detect the expression of the VSV-IN-G protein and VSV-NJ-G in rAds by western blotting as previously described [27].

For the IFA, Vero cells infected with rAd-IN-NJ were fixed with paraformaldehyde for 30 min at 4 °C. After washing three times with PBST, the cells were incubated with anti-VSV-IN-G protein mouse McAb (1:2000) or anti-VSV-NJ goat PcAb (1:500). Following fluorescein isothiocyanate (FITC) labeled goat anti-mouse IgG or FITC- rabbit anti-goat IgG were applied and incubated for 30 min at 37 °C. The cells were then washed three times and visualized under a fluorescent microscope.

### Immunization of mice and goat and sample collection

Mice: In order to evaluate the immune response to the rAds, the study selected 50 six-eight week-old female BALB/c mice. Mice were randomly divided into five groups, with 10 mice per group. The three groups of mice were respectively inoculated subcutaneously three times at two-week intervals with 10^8^ TCID_50_ rAd-IN, rAd-NJ, or rAd-IN-NJ. Two mice groups were respectively inoculated with the wild-type adenovirus and PBS, which were used as the negative control.

After 0, 2, 4, and 6 weeks of the first inoculation, blood was collected from the retrobulbar plexus of the mice. The serum was collected from the blood, incubated at 4 °C, and centrifuged at 4 °C (3000 rpm for 10 min). Then the serum was stored at − 20 °C for future use to detect specific antibody levels using a neutralization test. At the end of the experiments, the mice were euthanized using CO_2_ as previously described [28]. During the bleeding from retrobulbar plexus, the mice were anesthetized using phenobarbital.

Goats: 20 outbred healthy Boer goats (6 months old) were divided randomly into five groups, with four goats per group, and housed in separate rooms. All goats were negative for VSV infection as assessed by neutralizing antibodies (titers< 1:2). Groups 1, 2, and 3 were subcutaneously injected twice at three-week intervals with 10^8^ TCID_50_/mL rAd-IN, rAd-NJ, or rAd-IN-NJ. Groups 4 and 5 were inoculated subcutaneously with 10^8^ TCID_50_/mL wtAd or 1 mL PBS as negative controls. On 0, 3, 6, 9, and 12 weeks post-inoculation, blood was collected from the vein of each goat. Sera were collecting from the blood, incubated at 4 °C, and centrifuged at 4 °C (3000 rpm for 10 min). Then, the sera were stored at − 20 °C for future use to detect specific antibody levels using a neutralization test. At the end of the experiment, the goats were continued to feed.

### Determination of neutralizing antibody titers

Sera were collected and incubated at 56 °C for 30 min to inactivate complements. The sera were diluted five-fold with DMEM, and then serially diluted two-fold in DMEM. Serial dilutions of the serum were incubated with 200 TCID_50_ of wild-type viruses (VSV-IN or VSV-NJ) for 1 h at 37 °C. After incubation, samples were added to Vero cells in quadruplicate assays in 96-well plates, and incubated at 37 °C for 3 days. The CPE was observed and recorded 3 days after incubation. The VNA titer was defined as the highest serum dilution that inhibited CPE by at least 50%. A titer equivalent to 10 or higher was considered positive in this study.

### Lymphocyte proliferation assay

The lymphocyte proliferation assay for mice was conducted 2 weeks after the third immunization. Spleens were aseptically removed from three mice from each group and splenocyte suspensions were prepared. The splenocytes were extracted and purified using a Spleno-cyte Extraction kit (TBD Science, Tianjin, China) and seeded in 96-well flat-bottom plates at a density of 2 × 10^6^ cells per well in RPMI 1640 medium (Invitrogen, Grand Island, NY, USA) containing 10% FBS. Then, 100 μL of medium containing VSV-IN or VSV-NJ was added to each well of splenocytes. Concanavalin A (ConA) (Sigma Aldrich, St. Louis, MO, USA) was used as a positive control, and the medium was used as a negative control. The plates were incubated at 37 °C in 5% CO_2_ for 3 days. After 3 days, the proliferative response was determined using the Cell Titer 96 Aqueous One Solution Cell Proliferation kit (Promega, Madison, WI, USA), following the manufacturer’s instructions.

The lymphocyte proliferation assay for goats was conducted at week 9 after the first immunization. Blood was collected from the jugular vein of goats and was heparinized. Then, peripheral blood mononuclear cells (PBMC) were separated by Filoll-Hypque density gradient centrifugation (TBD Sciences, Tianjin, China), as described previously [27].

### Statistical analysis

The data generated in this study were analyzed using a one-way ANOVA method of the GraphPad Prism version 5.00 (GraphPad Software, San Diego, CA, USA). A *p* values less than 0.05 were considered statistically significant.

## Supplementary Information

**Additional file 1.**

## Data Availability

The datasets used and analyzed during the current study are available from the corresponding author on reasonable request.

## Abbreviations

CMV: Cytomegalovirus
CPE: Cytopathic effect
DIVA: Differentiating infected from vaccinated animals
DMEM: Dulbecco’s Modified Eagle’s medium
FITC: Fluorescein isothiocyanate
FBS: Fetal bovine serum
IFA: immunofluorescence assay
Kb: Kilobase
MOI: Multiplicity of infection
OIE: Office International des Epizooties, World Organization for Animal Health
PBMC: Peripheral blood mononuclear cell
PBS: Phosphate-buffered saline
rAd: Recombinant adenoviruses
RT-PCR: Reverse transcription-polymerase chain reaction
VNA: Virus-neutralizing antibody
VS: Vesicular stomatitis
VSV: Vesicular stomatitis virus
VSV-IN: Vesicular stomatitis virus-Indiana
VSV-IN-G: Vesicular stomatitis virus Indiana serotype glycoprotein
VSV-NJ: Vesicular stomatitis virus- New Jersey
VSV-NJ-G: Vesicular stomatitis virus New Jersey serotype glycoprotein
