# Supplementary material for: Recombinant adenovirus expressing vesicular stomatitis virus G proteins induce both humoral and cell-mediated immune responses in mice and goats
Source: BMC Vet Res. 2021 Jan 18;17:36. doi: 10.1186/s12917-020-02740-6 (PMC7814712; doi:10.1186/s12917-020-02740-6)
Supplement: Supplementary file 1 — Additional file 1. [file 12917_2020_2740_MOESM1_ESM.docx]

**Additional files:**

**Figure Legends**

**Figure 1 CPE of AAV-293 cells infected with the recombinant adenoviruses.** A: CPE of AAV-293 cells infected with the rAd-IN; B: CPE of AAV-293 cells infected with the rAd-NJ; C: CPE of AAV-293 cells infected with the rAd--IN-NJ; D: CPE of AAV-293 cells infected with the wtAd; E: CPE of AAV-293 cells infected with VSV; F: The normal AAV-293 cells.

**Figure 2 Identification of the recombinant adenovirus by PCR.** M: 2K Plus DNA Marker; 1: PCR product from rAd-IN; 2: PCR product from rAd-NJ; 3: PCR product from rAd-IN-NJ; 4: Negative control.

**Figure 3 PCR identification of different passages the recombinant adenovirus.** M:2K Plus DNA Marker; 1-4: PCR product from rAd-IN at passage 5th, 10th, 15th, 20th; 6-9: PCR product from rAd-NJ at passage 5th, 10th, 15th, 20th; 11-14: PCR product from rAd-IN-NJ at passage 5th, 10th, 15th, 20th; 5, 10, 15: Negative control.

**Figure 4 Expression of VSV G protein in the rAd-IN and rAd-NJ infected 293 cells by western blot.** M: Protein molecular weight Marker; 1: 293 cells infected with rAd-IN; 2, 3: 293 cells infected with wtAdV; 4: 293 cells infected with rAd-NJ.

**Figure 5 Expression of fusion protein in the rAd-IN-NJ infected 293 cells by western blot.** M: Protein molecular weight Marker; 2: 293 cells infected with rAd-IN-NJ (detected by VSV-IN monoclonal antibody); 3: 293 cells infected with rAd-IN-NJ (detected by VSV-NJ polyclonal antibody); 1, 4: Negative control.

**Figure 6 Identification of the expression of VSV G protein by IFA.** A: Vero cells infected with rAd-IN (detected by VSV-IN monoclonal antibody; B: Vero cells infected with rAd-NJ (detected by VSV-NJ polyclonal antibody); C: Vero cells control.

**Figure 7 Identification of expression of VSV fusion G protein in Vero cells by IFA.** A: Vero cells infected wit**Figure Legends**

**Figure 1 CPE of AAV-293 cells infected with the recombinant adenoviruses.** A: CPE of AAV-293 cells infected with rAd-IN; B: CPE of AAV-293 cells infected with rAd-NJ; C: CPE of AAV-293 cells infected with rAd-IN-NJ; D: CPE of AAV-293 cells infected with wtAd; E: CPE of AAV-293 cells infected with VSV-IN; F: Uninfected AAV-293 cells. CPE, cytopathic effect; rAd-IN, recombinant adenovirus-Indiana, rAd-NJ, recombinant adenovirus-New Jersey; rAd-IN-NJ, recombinant adenovirus-Indiana-New Jersey; wtAd, wild-type adenovirus; VSV-IN, vesicular stomatitis virus-Indiana

**Figure 2 Identification of the recombinant adenovirus by PCR.** M: 2K Plus DNA marker; 1: PCR product from rAd-IN; 2: PCR product from rAd-NJ; 3: PCR product from rAd-IN-NJ; 4: Negative control. rAd-IN, recombinant adenovirus-Indiana; rAd-NJ, recombinant adenovirus-New Jersey; rAd-IN-NJ, recombinant adenovirus-Indiana-New Jersey; PCR, polymerase chain reaction

**Figure 3 PCR identification of different passages the recombinant adenovirus.** M: 2K Plus DNA marker; 1-4: PCR product from rAd-IN at the 5th, 10th, 15th, and 20th passage; 6-9: PCR product from rAd-NJ at the 5th, 10th, 15th, and 20th passage; 11-14: PCR product from rAd-IN-NJ at the 5th, 10th, 15th, and 20th passage; 5, 10, 15: Negative control. rAd-IN, recombinant adenovirus-Indiana; rAd-NJ, recombinant adenovirus-New Jersey; rAd-IN-NJ, recombinant adenovirus-Indiana-New Jersey; PCR, polymerase chain reaction

**Figure 4 Identification of the G proteins of rAd-IN and rAd-NJ in** **AAV- 293 cells by western blot.** M: Protein molecular weight marker; 1: AAV-293 cells infected with rAd-IN (detected by the VSV-IN G monoclonal antibody); 2: AAV-293 cells infected with wtAdV (detected by the VSV-IN G monoclonal antibody); 3: AAV-293 cells infected with wtAdV (detected by the VSV-NJ polyclonal antibody); 4: AAV-293 cells infected with rAd-NJ (detected by the VSV-NJ polyclonal antibody). rAd-IN, recombinant adenovirus-Indiana; rAd-NJ, recombinant adenovirus-New Jersey; wtAd, wild-type adenovirus

**Figure 5 Identification of the fusion G protein of rAd-IN-NJ in AAV-293 cells using a Western blotting.** M: Protein molecular weight marker; 1: AAV-293 cells infected with wtAd (detected by the VSV-IN G monoclonal antibody); 2: AAV-293 cells infected with rAd-IN-NJ (detected by the VSV-IN G monoclonal antibody); 3: AAV-293 cells infected with rAd-IN-NJ (detected by the VSV-NJ polyclonal antibody); 4: AAV-293 cells infected with wtAd. wtAd, wild-type adenovirus; rAd-IN-NJ, recombinant adenovirus-Indiana-New Jersey

**Figure 6 Identification of the fusion G protein of rAd-IN-NJ in Vero cells using an immunofluorescence assay.** A: Vero cells infected with rAd-IN-NJ (detected by the VSV-IN monoclonal antibody); B: Vero cells infected with rAd-IN-NJ (detected by the VSV-NJ polyclonal antibody); C: Vero cells infected with wtAd control (detected by the VSV-IN monoclonal or VSV-NJ polyclonal antibody). rAd, recombinant adenovirus; wtAd, wild-type adenovirus; rAd-IN-NJ, recombinant adenovirus-Indiana-New Jersey; VSV-IN, vesicular stomatitis virus-Indiana; VSV-NJ, vesicular stomatitis virus-New Jersey

**Figure 7 Proliferation of splenocytes to virus stimulation in the immunized mice.**

rAd-IN, recombinant adenovirus-Indiana; rAd-NJ, recombinant adenovirus-New Jersey; rAd-IN-NJ, recombinant adenovirus-Indiana-New Jersey; wild-type adenovirus; PBS, phosphate-buffered saline; VSV-IN, vesicular stomatitis virus-Indiana; VSV-NJ,vesicular stomatitis virus-New Jersey

**Figure 8 Proliferation of peripheral blood mononuclear cells to virus stimulation in the immunized goats.** rAd-IN, recombinant adenovirus-Indiana; rAd-NJ, recombinant adenovirus-New Jersey; rAd-IN-NJ, recombinant adenovirus-Indiana-New Jersey; wild-type adenovirus; PBS, phosphate-buffered saline; VSV-IN, vesicular stomatitis virus-Indiana; VSV-NJ, vesicular stomatitis virus-New Jersey

**Tables**

**Table 1** Detection of VSV neutralizing antibodies in mice induced by recombinant adenovirus with VSV-IN at different time points

a. Mean neutralizing antibody titers at that time point (range of antibody titers). VSV-IN, vesicular stomatitis virus-Indiana; rAd-IN, recombinant adenovirus-Indiana; rAd-NJ, recombinant adenovirus-New Jersey; rAd-IN-NJ, recombinant adenovirus-Indiana-New Jersey; wtAd, wild-type adenovirus; PBS, phosphate-buffered saline

**Table 2** Detection of VSV neutralizing antibodies in goats induced by recombinant adenovirus with VSV-IN at different time points

a. Mean neutralizing antibody titers at that time point (range of antibody titers). VSV-IN, vesicular stomatitis virus-Indiana; rAd-IN, recombinant adenovirus-Indiana; rAd-NJ, recombinant adenovirus-New Jersey; rAd-IN-NJ, recombinant adenovirus-Indiana-New Jersey; wtAd, wild-type adenovirus; PBS, phosphate-buffered saline

**Table 3** Detection of VSV neutralizing antibodies in mice induced by recombinant adenovirus with VSV-NJ at different time points

a. Mean neutralizing antibody titers at that time point (range of antibody titers). VSV-NJ, vesicular stomatitis virus-New Jersey; rAd-IN, recombinant adenovirus-Indiana; rAd-NJ, recombinant adenovirus-New Jersey; rAd-IN-NJ, recombinant adenovirus-Indiana-New Jersey; wtAd, wild-type adenovirus; PBS, phosphate-buffered saline

**Table 4** Detection of VSV neutralizing antibodies in goats induced by recombinant adenovirus with VSV-NJ at different time points

a. Mean neutralizing antibody titers at that time point (range of antibody titers). VSV-NJ, vesicular stomatitis virus-New Jersey; rAd-IN, recombinant adenovirus-Indiana; rAd-NJ, recombinant adenovirus-New Jersey; rAd-IN-NJ, recombinant adenovirus-Indiana-New Jersey; wtAd, wild-type adenovirus; PBS, phosphate-buffered saline
